# Anticorrosion potential of a bioemulsifier produced by *Psychrobacillus antarcticus* Val9 isolated from Antarctic soil

**DOI:** 10.3389/fmicb.2025.1694832

**Published:** 2026-01-05

**Authors:** Lívia Vieira Araujo de Castilho, Aline Loureiro Barreto, Karen Caroline Ferreira Santaren, Mariana Barbalho Farias Rosenblatt, Igor Taveira, Jefferson Cypriano, Fernanda Abreu, Mateus Gomes de Godoy, Diogo de Azevedo Jurelevicius, Lucy Seldin

**Affiliations:** 1 Laboratório de Genética Microbiana, Instituto de Microbiologia Paulo Góes, Centro de Ciências da Saúde, Universidade Federal do Rio de Janeiro, Rio de Janeiro, Rio de Janeiro, Brazil; 2 Laboratório de Biologia Celular e Magnetotaxia, Instituto de Microbiologia Paulo Góes, Centro de Ciências da Saúde, Universidade Federal do Rio de Janeiro, Rio de Janeiro, Rio de Janeiro, Brazil; 3 Unidade de Microscopia Multiusuário, Instituto de Microbiologia Paulo Góes, Centro de Ciências da Saúde, Universidade Federal do Rio de Janeiro, Rio de Janeiro, Rio de Janeiro, Brazil; 4 Laboratório de Ecologia e Biotecnologia Microbiana, Instituto de Microbiologia Paulo Góes, Centro de Ciências da Saúde, Universidade Federal do Rio de Janeiro, Rio de Janeiro, Rio de Janeiro, Brazil

**Keywords:** bioemulsifier, *Psychrobacillus antarcticus*, microbiologically influenced corrosion (MIC), anticorrosion, antibiofilm

## Abstract

**Introduction:**

Microbiologically influenced corrosion (MIC) poses a persistent challenge in industrial systems, particularly in oilfield infrastructure, where biofilm-forming microorganisms accelerate metal degradation. This study evaluated the anticorrosive, antibiofilm, and biocidal properties of two bioproducts—a bioemulsifier from *Psychrobacillus antarcticus* Val9 and a surfactin from Bacillus velezensis H2O-1— simulating water injection header conditions.

**Methods:**

The bioproducts were produced and characterized through emulsification indices, surface tension, and oil displacement assays. Their protective effects against biocorrosion were assessed via carbon steel mass loss, scanning electron microscopy, epifluorescence microscopy, surface roughness analysis, ATP quantification, and molecular profiling of microbial communities.

**Results and Discussion:**

The compounds demonstrated desirable physicochemical properties and maintained stability under the tested conditions. All the treatments significantly reduced the carbon steel mass loss over 96 h, with protection rates ranging from 58.2% ± 11.3 to 94.6% ± 2.0. Microscopic analyses revealed diminished biofilm roughness and disrupted extracellular matrix cohesion, indicating impaired biofilm maturation. ATP assays and qPCR data revealed selective microbial suppression without triggering metabolic rebound, suggesting the destabilization of biofilm homeostasis. Furthermore, 16S rRNA gene sequencing and absolute quantification revealed a shift in the microbial community structure, with a reduced abundance of corrosion-associated taxa and enrichment of fewer metal-aggressive genera.

**Conclusion:**

These findings highlight the dual action of the tested bioproducts: direct surface protection and strategic microbial community modulation. This integrated approach offers a sustainable alternative to conventional chemical biocides, with potential applications in offshore pipelines and industrial water systems.

## 1 Introduction

Microbiologically influenced corrosion (MIC) is defined as the loss of metal caused by the presence and metabolic activity of microorganisms (Senthilmurugan et al., 2021). It affects fossil fuel recovery, transport, and storage processes, generating a significant economic impact due to equipment maintenance, and depending on the extent and severity of corrosion, it may lead to environmental damage—posing a major challenge for industries in this sector (Giorgi-Pérez et al., 2021; Senthilmurugan et al., 2021).

Bacterial growth on diverse surfaces leads to biofilm formation, which impairs heat transfer and accelerates metal corrosion. Within biofilms, microbes produce extracellular polymeric substances (EPSs) that facilitate attachment, regulate gas diffusion and pH, and absorb nutrients. Acting as both the anode and cathode, the bacteria generate a potential difference that drives and intensifies the corrosion process (Vignesh et al., 2025a).

Microbial activity is considered one of the primary causes of water quality deterioration and biofouling in water production and injection systems. MIC is predominantly caused by sulfate-reducing bacteria (SRB), which are anaerobes capable of using different terminal electron acceptors in respiration and corrosion processes. Their peak activity occurs during biofilm formation (Giorgi-Pérez et al., 2021). Furthermore, studies have indicated the involvement of other microorganisms, such as thiosulfate-reducing bacteria, sulfur-oxidizing bacteria (SOB), and metal-oxidizing bacteria (MOB), which include iron- and manganese-oxidizing bacteria, metal-reducing bacteria (MRB), sulfate-reducing archaea, methanogenic archaea, and other microorganisms that secrete organic acids and/or produce EPSs (Senthilmurugan et al., 2021; Liu et al., 2023).

Biofilm formation induces and accelerates the MIC process through synergistic interactions among diverse microorganisms that act collectively on metal surfaces. Systems characterized by complex microbial communities—such as the water injection header used for oil recovery—present a higher MIC risk than those with low microbial diversity (Senthilmurugan et al., 2021). Compared with single-species communities, these complex microbial communities combine synergistic and competitive interactions, intensifying the MIC effect (Liu et al., 2023). Given the widespread use of steel alloys in the oil industry, thoroughly investigating corrosion damage mechanisms, MIC-related byproducts, and microbial communities involved is vital (Giorgi-Pérez et al., 2021; Liu et al., 2023).

Understanding microbial behavior and the commonly associated microbial succession in these communities will aid in the development of new strategies to control MIC. These strategies are usually focused on surface treatment (physical and cathodic protection), coating, material design (antibacterial elements and texture tuning), biological protection (EPS protection, antimicrobial substances, and biomineralization), and other methods (Liu et al., 2023). For example, Vignesh et al. (2024) investigated the synthesis of an N-substituted tetrabromophthalic compound as a corrosion inhibitor and its effectiveness against MIC caused by *P. aeruginosa* in a cooling water system on carbon steel. Their study demonstrated a reduction in corrosion rates and suppression of biofilm formation.

Currently, most microbial control agents used in water injection systems on oil platforms are chlorine-based biocides, 2,2-dibromo-3-nitrilopropionamide (DBNPA), tetrakis(hydroxymethyl)phosphonium sulfate (THPS), and glutaraldehyde, which are chemical, non-biodegradable, and in many cases exacerbate corrosion due to incompatibility with the metal alloys employed (Ganie and Mady, 2025). Therefore, the development of alternative solutions that are environmentally safe, derived from renewable sources, and simultaneously exhibit biocidal properties and compatibility with metal alloys, even being able to adsorb to the surface, forming a protective film to prevent biofilm formation and therefore biocorrosion, is crucial for helping the oil industry overcome this persistent challenge (Rocha et al., 2021; Ganie and Mady, 2025; Vignesh et al., 2025b).

*Psychrobacillus antarcticus* strain Val9 is a gram-positive, motile, psychrotolerant, and aerobic bacterium isolated from Antarctic soil on King George Island. The strain grows optimally at 15 °C, pH 8, and 3% NaCl, and produces a bioemulsifier with potential biotechnological applications (Vollú et al., 2014). Its ability to produce a bioemulsifier under low-temperature conditions suggests potential applications in bioremediation and corrosion mitigation in cold environments (da Silva et al., 2023). If Val9 is capable of promoting the passivation of metal alloys or altering the microbial community responsible for the MIC process, it could help prevent or delay corrosion, resulting in economic benefits and reduced environmental impact.

Similarly, the *Bacillus velezensis* strain H2O-1 was isolated, identified, and described, and its bioproduct was characterized by our group. This strain is a gram-positive, spore-forming bacterium that produces biosurfactants with remarkable surface-active and emulsifying properties. *Bacillus velezensis* strain H2O-1 synthesizes five surfactin homologs with fatty acid chains ranging from C11 to C16 and is capable of effectively reducing surface and interfacial tension. Notably, its efficacy is maintained under operational environmental conditions such as elevated pressure, temperature, and salinity (Korenblum et al., 2005, 2012; Guimarães et al., 2019, 2021; de Paula Vieira de Castro et al., 2022).

The objective of this study was to evaluate the potential use of these two biotechnological products—individually and in combination—in injection header water to protect carbon steel alloy surfaces commonly used in the oil industry against MIC. To this end, their biocidal, antibiofilm, and anticorrosive properties were assessed, along with their effects on the microbial community dynamics present in the injection header water. It is anticipated that the application of these bioproducts, either alone or synergistically, may enhance corrosion mitigation associated with MIC. This approach could enable the partial or complete replacement of conventional chemical biocides with environmentally benign alternatives that are nonaggressive to metal surfaces.

## 2 Materials and methods

### 2.1 Bioproducts formulation and production

#### 2.1.1 Bioemulsifier

The bioemulsifier was produced by the strain of *Psychrobacillus antarcticus* Val9 (stored at the culture collection of the “Laboratório de Genética Microbiana, UFRJ,” Rio de Janeiro, Brazil), as described in da Silva et al. (2023). The strain was first cultured on tryptic soy agar (TSA) for 48 h at 28 °C for reactivation, since it was stored at −80 °C. Following colony scraping with sterile saline (0.85% NaCl), the suspension was standardized by optical density at 600 nm and inoculated (10%) into the production medium (TSB). The cultures were incubated at 15 °C for 96 h at 150 rpm. Optical density measurements were correlated with colony-forming unit (CFU/mL) counts via the spread plate technique.

Bioemulsifier production was assessed via the emulsification index (E_24_) against n-hexadecane (see Section 2.2.2). After cell removal by centrifugation at 8,000 rpm and 10 °C (Beckman Coulter Avanti J-E), the bioemulsifier was recovered via ethanol precipitation (2:1) at 4 °C for 48 h. The resulting precipitate was centrifuged and washed three times with distilled water prior to use (da Silva et al., 2023; Yadav et al., 2024).

#### 2.1.2 Surfactin

Surfactin was produced by the strain *Bacillus velezensis* H2O-1 (“Laboratório de Genética Microbiana,” UFRJ, Rio de Janeiro, Brazil), which was previously isolated, identified, and characterized by our group (Korenblum et al., 2005, 2012). The strain was first cultured on LB agar for 48 h at 30 °C, since it was stored at −80 °C. Surfactin production, purification, and quantification were performed as described by Guimarães and coworkers (Guimarães et al., 2019), using a mineral medium containing (w/v) NaCl 1.0, Na_2_HPO_4_ 0.5, KH_2_PO_4_ 0.2, MgSO_4_ 0.02, and (NH_4_)_2_SO_4_ 0.2, with glucose (1.0% w/v) as the sole carbon source. The cultures were incubated for 72 h at 30 °C and 170 rpm.

Following cell removal by centrifugation, the supernatant was lyophilized and stored until further use. Surfactin quantification was performed by injecting purified samples into a high-performance liquid chromatography (HPLC) system (Agilent Technologies 1,200, Santa Clara, CA, United States) equipped with a UV detector (210 nm) and a ZORBAX C18 column (150 × 4.6 mm, 5-μm particle size; Agilent Technologies, Santa Clara, CA, United States). The peaks corresponding to the distinct isoforms of surfactin were integrated and quantified according to the calibration curve obtained using the Sigma–Aldrich surfactin standard (≥98% purity, Ref: S3523) (Guimarães et al., 2019).

### 2.2 Physicochemical characterization of bioproducts

#### 2.2.1 Surface tension (SFT)

The pendant drop method (Song and Springer, 1996) was used to assess the SFT, interfacial tension (IFT) (against n-hexadecane), and critical micelle concentration (CMC) from the bioproducts using a Krüss DSA100 goniometer (Model OF 3210). The diameter of the needle used was measured with a calibrated micrometer to adjust the imaging zoom with the real needle value through the software Krüss Advance (Kruss, Germany). The droplet formation speed was set at 16 μL/s using the motorized system controlled by the same software. The calibration and monitoring of the goniometer were performed with deionized water, considering temperature and atmospheric pressure conditions. Measurements were performed at 23 °C with a controlled relative humidity of 55%, and the results represent the average of at least three pendant drops. The same technique was employed to determine the CMC across serial dilutions of the biosurfactant, in accordance with Sheppard and Mulligan (Sheppard and Mulligan, 1987). Dynamic surface tension (DSFT) was evaluated by averaging at least three drops, monitored from their initial formation until stabilization of the surface tension (2 min), and measurements were recorded at 5-s intervals, totaling 25 measurements for each drop.

#### 2.2.2 Emulsification index (E_24_)

Emulsification measurements were performed via the addition of equal volumes of hydrocarbons (*n-*hexadecane, sunflower oil, extra virgin olive oil, kerosene, or *Lippia gracilis* essential oil) and the bioproduct (bioemulsifier or surfactin) to 2.0 mL microtubes following vortexing for 2 min. The emulsification index was determined after 24 h of static incubation at room temperature and was calculated by dividing the height of the emulsion layer by the total height of the mixture and multiplying the result by 100 (Cooper, 1986; Lamilla et al., 2018). For stability assessments, emulsions were maintained at room temperature, and the emulsion layer height was monitored over a 30-day period. The emulsification stability was further evaluated at various pH values (2, 4, 7, 10, and 12), salinities (3, 6, 9, and 12% NaCl), and temperatures (−20, 5, 15, 23, 100, and 121 °C).

#### 2.2.3 Oil displacement test

The diameters (in mm) of the halos formed after dispensing 10 μL of the bioemulsifier/biosurfactant onto a crude oil layer (20 μL on a Petri dish containing 50 mL of distilled water) were measured. Sodium dodecyl sulfate (SDS) and uninoculated culture media were used as positive and negative controls, respectively (Alvarez et al., 2015).

### 2.3 Microcosm simulation and evaluation of the effects of bioproducts

#### 2.3.1 Biofilm bioreactor setup and operation

To evaluate the antimicrobial, antibiofilm, and anticorrosive efficiency of the selected products, custom-designed bioreactors based on previous work (Rocha et al., 2021) were constructed as follows: jacketed borosilicate glass bioreactors—each with a working volume of approximately 1 L—were coupled in series to enable internal temperature control via a recirculation system connected to an external water bath. Internal agitation was provided via a magnetic stirring platform. A lateral outlet for bulk disposal was used to level the internal volume of the reactors, which was connected by tubing to a 3 L Erlenmeyer flask. This outlet was sealed after leveling. The bioreactor lids included openings fitted with rods to support triplicate samples of metal coupons (carbon steel). These coupons remained fully submerged, exposing both sides of the samples to the reactive medium. Additional ports were used for nitrogen injection (to maintain a negative redox potential), sample collection, periodic administration of the test products, and gas venting. The gas outlet was coupled to a scrubber containing a neutralizing solution designed to mitigate the potential release of hydrogen sulfide (H_2_S). The bioreactor system is described in Supplementary Figure S1. Prior to use, the bioreactor assembly was sterilized by autoclaving at 121 °C and 1 atm for 20 min.

In accordance with Rocha et al. (2021), the bioreactors were maintained under continuous nitrogen purging coupled to a sterile PTFE membrane (0.22 μm, TuttNauer). The water from the injection header was transferred aseptically using a sterile syringe under laminar flow. Additionally, bioproducts injections and sample collection were performed with sterile syringes via the injection/collection hose in the bioreactor lid. The temperature was maintained at 38.4 °C with constant agitation at 60 rpm for 14 days to allow for the stabilization of biofilm formation on the carbon steel coupons (monitored by ATP and microscopy analysis). Bulk fluid containing native microorganisms from a water-injection header system was used. After this period, the first injection of test products—bioemulsifier, biosurfactant, or a mixture of both—was performed at a concentration of 200 ppm. One bioreactor was maintained as a control. Furthermore, 48 h after the initial injection, samples of the bulk and coupons were collected for microscopic analysis, ATP quantification, coupon mass loss, and DNA extraction for qPCR and sequencing. A second injection of the test products was subsequently administered, followed by a second round of sampling 48 h later. The 48-h interval between each injection and the chosen concentration was based on established oil industry practices with commercial chemical biocides.

#### 2.3.2 Coupon analysis

##### 2.3.2.1 Preparation and characteristics of the metallic coupons

Metallic carbon steel coupons (surface area: 3.5 cm^2^), which were used as test samples for evaluating biofilm formation and corrosion, were selected because of their relevance as one of the most commonly employed alloys in the oil industry. These coupons followed the USI-SAC 350 specifications, with the chemical composition as follows: maximum C, 0.18%; maximum Mn, 1.40%; Si, 0.5–1.5%; P, 0.010–0.060%; and maximum S, 0.030%. The mechanical properties are yield strength (YS) ≥ 350 MPa, tensile strength (TS) between 500 and 600 MPa, and a thickness of 1.03 mm (Quispe et al., 2022).

The carbon steel coupons underwent a cleaning protocol comprising immersion in 99% (v/v) ethanol for 10 min in an ultrasonic bath, rinsing with distilled water, immersion in a 2% aqueous solution of commercial detergent followed by another 10 min in an ultrasonic bath, a second rinse with distilled water, immersion in acetone, and drying in a fume hood until complete solvent evaporation. The samples were then used for subsequent analyses (Nitschke et al., 2009).

##### 2.3.2.2 Coupon loss of mass (corrosion effect)

Mass loss was determined by subtracting the initial weight of the coupons at time zero (start of the experiment) from the weight measured at the evaluated time points (48 and 96 h) after a standardized cleaning procedure to remove biofilms by immersion in 18% (v/v) hydrochloric acid, neutralization in saturated sodium bicarbonate solution, rinsing with distilled water, immersion in acetone, and drying in a fume hood until a constant weight was reached (Nemati et al., 2001).

#### 2.3.3 Scanning electron microscopy (SEM)

For SEM, samples were fixed in 2.5% glutaraldehyde solution prepared in 0.1 M sodium cacodylate buffer (pH 7.2) for 24 h and kept refrigerated at 10 °C. After fixation, the samples were washed three times with the same buffer for 15 min per wash. The postfixation step using osmium tetroxide was omitted. Dehydration was carried out via the addition of ethanol solutions of increasing concentrations (30, 50, 70, 90%—each applied once; and 100%—applied three times), with a 15-min incubation at each stage. The samples were subsequently treated with a 1:1 solution of ethanol and hexamethyldisilazane (HMDS) for 5 min, followed by the addition of pure HMDS for another 5 min. After drying, the samples were mounted on metal supports with carbon tape and coated with gold in a Leica EM SCD 050 sputter coater (Leica Microsystems, Germany). Finally, the samples were examined via a Quattro S scanning electron microscope (Thermo Fisher Scientific, United States) operating at an accelerating voltage of 5 kV.

#### 2.3.4 Epifluorescence microscopy for determining cell viability (biofilm and planktonic)

Biofilm viability was assessed via the LIVE/DEAD™ Bacterial Viability kit (Thermo Fisher Scientific, USA) following the manufacturer’s protocol with slight modifications. First, the metal coupons containing the biofilms were stained with LIVE/DEAD™ reagent for 10 min at room temperature, avoiding direct light exposure. After staining, the samples were again rinsed twice with artificial seawater (ASW) to remove excess dye. The biofilms were then fixed in 4% formaldehyde for 15 min at room temperature. After fixation, the samples were rinsed twice with ASW to remove residual formaldehyde. To preserve the structural integrity of the biofilm for imaging, the samples were stabilized via a 1% agarose matrix.

Epifluorescence images were acquired via a Zeiss Axio Imager D2 microscope (Zeiss, Germany). The fluorescence visualization employed two filter sets: Filter Set 10, with bandpass (BP) excitation at 450–490 nm, a beam splitter (FT) at 510 nm, and longpass (LP) emission from 515 nm; and Filter Set 00, with BP excitation at 546/12 nm, FT at 560 nm, and BP emission between 575and 640 nm. These parameters enabled the precise visualization of stained cellular structures, complemented by morphological analysis via differential interference contrast (DIC).

#### 2.3.5 Surface roughness

The captured images were subjected to quantitative analysis via ImageJ software with the “Waveness and Roughness” plugin, with a focus on surface roughness evaluation and five independent replicates for each experimental condition (*n* = 5). Representative areas were selected from the images for analysis, avoiding regions containing artifacts or irregularities unrelated to the biofilm. The images were initially converted to 8-bit grayscale, and topographic profiles were generated on the basis of height variations observed in the images. The mean and standard deviation values were extracted automatically. Additionally, three-dimensional (3D) maps were generated to visualize detailed differences in surface roughness among the tested conditions. These quantitative and qualitative data were used to characterize structural changes in the biofilm in response to the various treatments applied. For statistical inference, samples were tested for normality via the Shapiro–Wilk test and fitted with a Gaussian distribution with analysis of the coefficient of determination (i.e., *R*^2^). One-way ANOVA followed by Tukey’s multiple comparisons test was subsequently performed between the control and treatment conditions.

#### 2.3.6 ATP determination (biofilms and planktonic bacteria)

ATP determination was performed in both the bulk (planktonic) and the biofilms formed on the coupons collected from the bioreactors at the evaluated time points (0, 48, and 96 h). The Qiagen PhotonMaster Reagent kit (QIAGEN GmbH, Hilden, Germany) and DSA PhotonMaster Reagent kit (LuminUltra Technologies Ltd., Fredericton, New Brunswick, Canada) were used according to the manufacturer’s protocols. Readings were taken via the PhotonMaster Luminometer & Bluetooth Module (Lumin Ultra Technologies Ltd., New Brunswick, Canada).

#### 2.3.7 Molecular analyses for microbial community quantification and characterization

##### 2.3.7.1 Total DNA extraction

The collected samples (biofilm and planktonic) were processed for total DNA extraction using the FastDNA Spin kit for Soil (MP Biomedicals, California, United States). The biofilm material was scraped from the surface and transferred to sterile tubes, while 50 mL of the planktonic suspension was collected and centrifuged to pellet the cells. In each case, the material was transferred to an extraction tube, and the DNA was isolated according to the manufacturer’s instructions. DNA quantity and quality were evaluated via a Qubit 3.0 fluorometer (Thermo Fisher Scientific, United States). The extracted DNA was used for qPCR-based absolute microbial quantification, and the structure and composition of the microbial community were determined through 16S rRNA gene sequencing, which was performed as follows.

##### 2.3.7.2 Quantitative PCR (qPCR)

The absolute abundance of microorganisms in each sample was quantified via qPCR targeting the 16S rRNA gene. The universal primers 341F/806R were used for amplification (Caporaso et al., 2011, 2012). Microbial quantification was performed via a Rotor-Gene 6,000 thermal cycler (Corbett Life Science, Australia). A standard curve for 16S rRNA copies was generated as described in Prediger (2013) via serial dilutions of *Pseudomonas aeruginosa* GS1 DNA corresponding to 10^8^–10^2^ copies of the 16S rRNA gene per μL.

To compare the abundance of prokaryotic cells observed at each sampling point (when replicate samples were available), the qPCR data distributions were first tested for normality (Shapiro–Wilk test) and homoscedasticity (Levene’s test). When assumptions were met, significant differences among samples were evaluated by ANOVA followed by Tukey’s *post hoc* test. When assumptions were not met, differences were assessed via the Kruskal–Wallis test (*p* < 0.05).

##### 2.3.7.3 Microbial 16S rRNA gene sequencing

To analyze the structure and composition of the microbial community, sequencing of the gene encoding 16S rRNA was performed via the Illumina MiSeq platform^1^. The universal primers 341F/806R were used for amplification (Caporaso et al., 2011, 2012), following the protocols described by the Earth Microbiome Project^2^ and Thompson et al. (2017). The sequences obtained were demultiplexed and analyzed via the QIIME2 pipeline, version 2020.2 (Bolyen et al., 2019). Briefly, the Deblur tool (Amir et al., 2017) was used to denoise the sequences, assess sequence quality—remove low-quality reads and potential chimeras—and generate amplicon sequence variants (ASVs) with a distribution table of ASVs per sample. Multiple sequence alignments of ASVs were then performed via MAFFT, and a phylogenetic tree was constructed via FastTree. The ASV table, which was normalized by rarefaction to an equal sequencing depth across samples, was used together with the phylogenetic tree to assess the alpha and beta diversity of the microbial communities.

The alpha diversity of each sample was estimated via observed ASV counts, the Shannon diversity index (Shannon and Weaver, 1949), and phylogenetic diversity (Faith and Baker, 2006). Alpha diversity metrics were compared across all samples via the Kruskal–Wallis test, with *p*-values adjusted via Benjamini–Hochberg (BH) false discovery rate (FDR) correction. Beta diversity analyses were performed via unweighted and weighted UniFrac metric distances and visualized via principal coordinate analysis (PCoA). Finally, a permutational multivariate analysis of variance (PERMANOVA) (Anderson, 2017) was performed to statistically compare the microbial community structure and composition observed in each sample.

##### 2.3.7.4 Taxonomic composition of the microbial community and determination of the absolute abundance of microbial groups

The taxonomy for each ASV was assigned via the SILVA 138 database (Quast et al., 2012) with a pretrained naive Bayes classifier implemented through the q2-feature-classifier command in QIIME2. To estimate the absolute abundance of microbial groups present in each sample, qPCR-derived values for total prokaryote quantification (bacteria/archaea) were considered the total number of bacterial and archaeal cells per sample, following the approach proposed by Morton et al. (2019). The relative abundance values for each microbial taxon—determined from sequencing analysis—were subsequently treated as the fractional representation of each microbial group within a given sample, as described by Jian et al. (2020). These relative abundances were converted into estimated absolute abundances by multiplying the relative abundance of each taxon by the total prokaryote count (qPCR result) for the corresponding sample, and Log₂ fold changes were calculated from the mean of two replicates (48 and 96 h), with a pseudocount of 0.01 applied to zeros; errors were estimated by standard deviation propagation.

## 3 Results

### 3.1 Production of bioproducts and their physicochemical properties

#### 3.1.1 Surfactin

The surfactin produced by *Bacillus velezensis* H2O-1 reached a yield of 200 mg/L, with the lyophilized product exhibiting 7% purity. The physicochemical properties were evaluated, resulting in an E_24_ of 47 ± 3.0%, a CMC of 30 mg/L, an SFT of 28.91 ± 0.2 mN/m, and positive results in both the oil displacement and drop collapse assays.

#### 3.1.2 Bioemulsifier

The bioemulsifier produced by *Psychrobacillus antarcticus* Val9 presented the highest yields at 72 and 96 h when cultivated at 15 °C, reaching emulsification index values of 61 ± 1.8% and 63 ± 1.7%, respectively. Interestingly, although strain Val9 exhibited the highest growth rate at 28 °C, the emulsification index at this temperature was markedly lower, reaching only 33.4 ± 3.4% after 48 h. Regarding its physicochemical properties, the bioemulsifier demonstrated a surface tension of 45.23 ± 1.6 mN/m, a positive response in the oil displacement test, and a negative result in the drop collapse assay.

To further characterize the emulsifying capacity of this bioproduct, various hydrocarbons were tested. Stable emulsions were achieved with all samples, with emulsification indices of 65.4 ± 2.07% for sunflower oil, 57.33 ± 0.25% for extra virgin olive oil, 55.23 ± 1.67% for kerosene, and 52.26 ± 0.80% for *Lippia gracilis* essential oil. The emulsion stability did not differ significantly across salt concentrations ranging from 3 to 6% (*p* ≤ 0.05), with values reaching 51.42 ± 2.2%. At higher concentrations (9 and 12%), the emulsifying activity decreased to 42.85 ± 4.94% but remained stable. The pH stability of the emulsifying activity was evaluated, and the results indicated no significant differences between pH 7 and pH 2, with values reaching approximately 60 ± 4.36%. However, increasing the pH to 10 and 12 resulted in a reduction in the emulsion layer to 48.5 ± 3.3% and 51.42 ± 3.3%, respectively, with no significant difference observed between these two conditions.

The influence of low temperatures (−20, 5, and 15 °C) on emulsifying performance was assessed over time, and no significant differences were observed relative to the control. In contrast, thermal treatment positively impacted the emulsifying ability: heating the cell-free broth at 100 °C for 30 min increased the emulsification index to 73.3 ± 1.3%, and autoclaving at 121 °C and 1 atm for 20 min further increased it to 76 ± 2.3%. A similar improvement was noted following product purification.

In the oil displacement test, the cell-free broth initially exhibited a negative result. However, following thermal treatments and purification, its ability to displace and emulsify petroleum improved notably—producing cloud-like formations that led to reclassification as a positive test.

**Figure 1:**
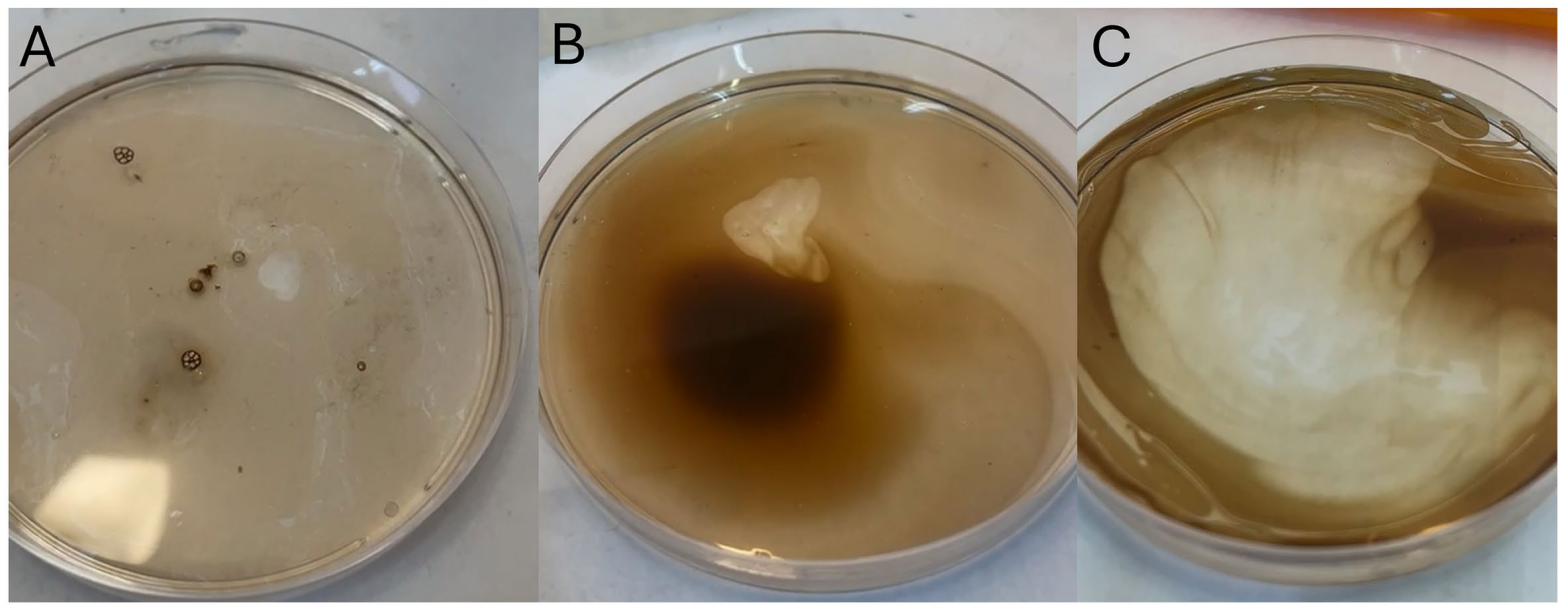
Oil displacement test of the bioemulsifier showing minimum halo formation with the cell-free broth **(A)**, an increase in halo formation after heating by autoclaving (121 °C, 1 atm, 20 min) **(B)**, and cloud-like halo formation throughout the oil layer after autoclaving and subsequent purification **(C)**.

Importantly, this outcome diverged from the classical oil displacement response, which typically presents as rapid halo formation. Instead, we observed a gradual development of halos resembling clouds (Figure 1), indicating a distinct emulsification dynamic.

### 3.2 Effect of the bioproducts on microcosms simulating the water injection header system

**Figure 2:**
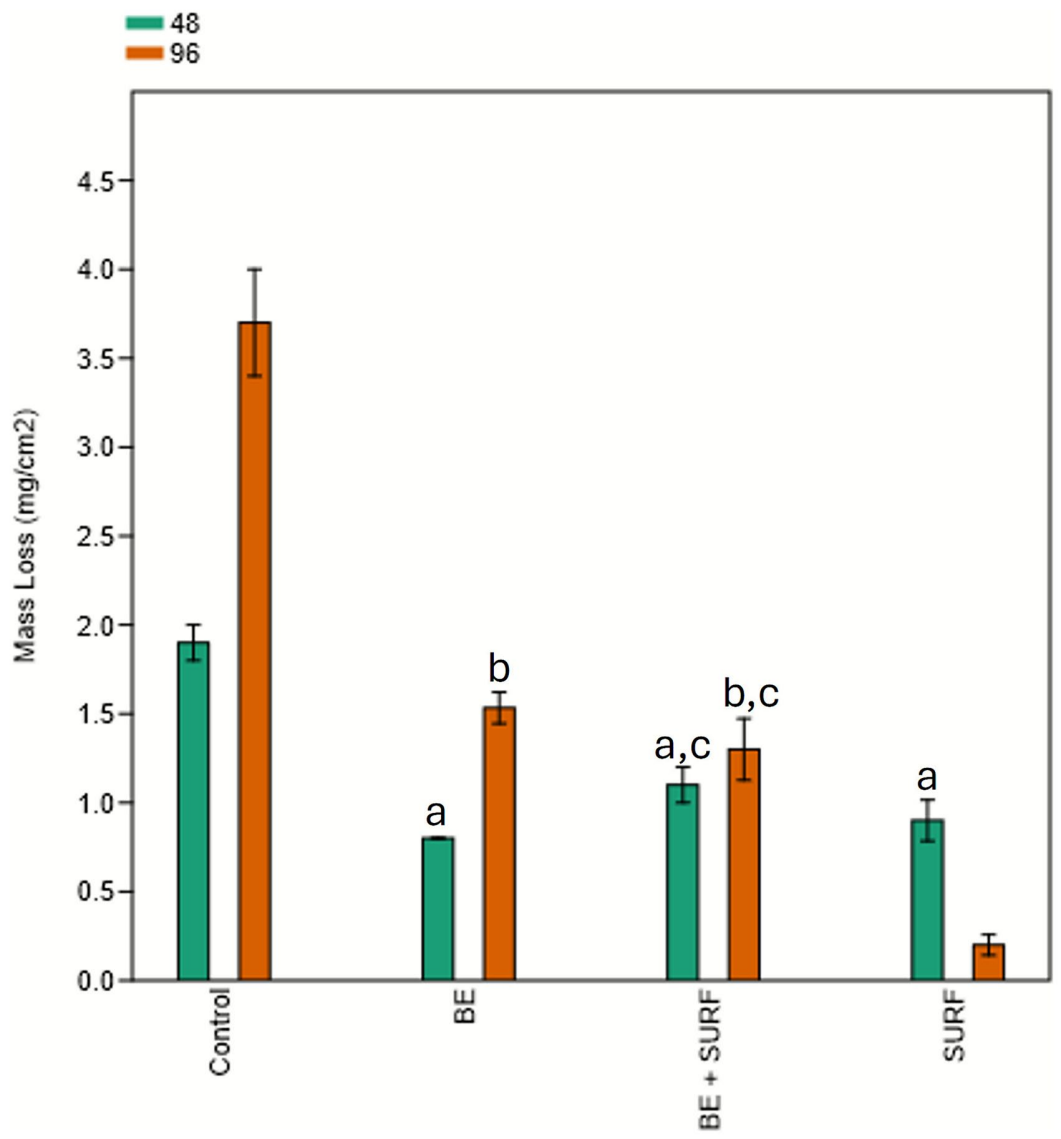
Mass loss of carbon steel coupons (mg/cm^2^) over time (48 and 96 h) under different treatment conditions: bioemulsifier (BE), surfactin (SURF), bioemulsifier + surfactin (BE+SURF), and control. Treatments labeled with the same letter do not differ statistically (*p* ≤ 0.05).

**Figure 3:**
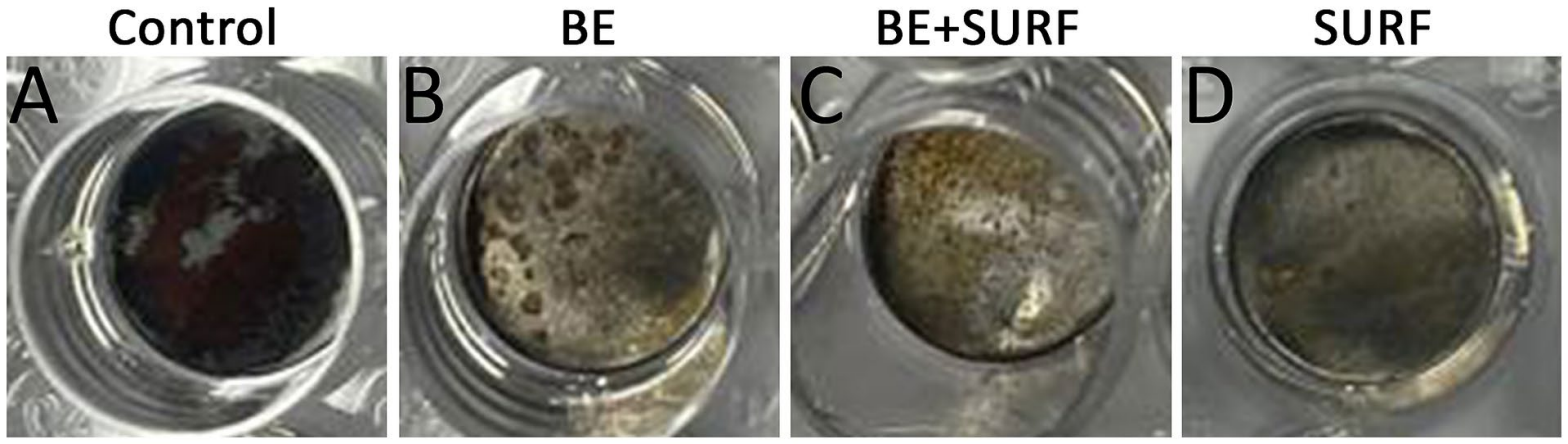
Photographs from the visual inspection of metallic surfaces show varying degrees of corrosion. The untreated sample **(A)** exhibits extensive damage, while samples treated with bioemulsifier **(B)**, bioemulsifier combined with surfactin **(C)**, and surfactin alone **(D)** demonstrate progressively enhanced protection. Notably, the surface treated with surfactin alone **(D)** shows the least corrosion, highlighting its superior effectiveness when added to injection header water.

Figure 2 presents the mass loss results of the carbon steel samples. Compared with the control treatment, the addition of the bioemulsifier, surfactin, or their combination into the water of the injection header system significantly reduced the mass loss of the carbon steel coupons. Within the first 48 h, no statistically significant differences were observed among the treatments. However, after 96 h, the most effective treatment was surfactin alone. Compared with individual applications, the combination of the bioemulsifier and surfactin did not have a synergistic effect on reducing mass loss. There was no significant difference between the bioemulsifier and the bioemulsifier combined with surfactin, although both treatments were significantly effective in protecting the surface relative to the control. The anticorrosive effect is also noticeable and clearly evident through simple visual inspection of the test samples, as shown in Figure 3, where all treated samples exhibit more intact surfaces than the control.

**Figure 4:**
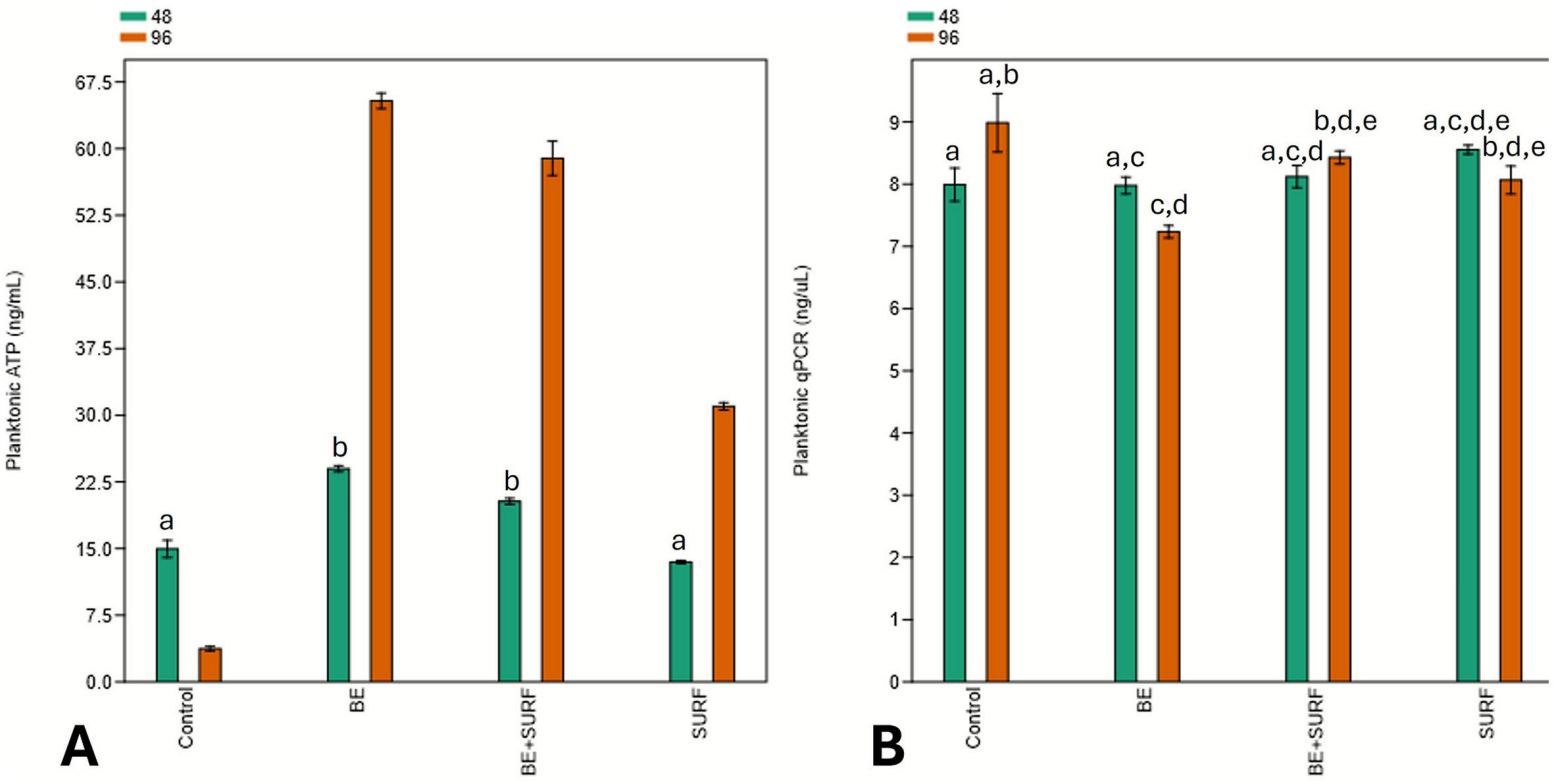
Planktonic ATP (ng/mL) **(A)** and cellular concentration (ng/μL) **(B)** over time (48 and 96 h) under different treatment conditions: bioemulsifier (BE), surfactin (SURF), bioemulsifier + surfactin (BE+SURF), and control. Treatments labeled with the same letter do not differ statistically (*p* ≤ 0.05).

The ATP analysis of the planktonic phase revealed an increase in the metabolic activity of the microorganisms over time across all the treatments compared with that of the control (Figure 4A). The exception was surfactin, which was not significantly different from the control at 48 h. The treatments with the bioemulsifier alone and with the bioemulsifier combined with surfactin were statistically equivalent during the first 48 h. Although a statistically significant difference in cellular metabolism was observed, the total number of cells verified by qPCR did not differ significantly across most treatments over time, as shown in Figure 4B. Differences were detected only between the control group and the bioemulsifier + surfactin treatment group compared with the bioemulsifier alone group at 96 h. The bioemulsifier treatment was able to reduce the number of cells, indicating that biocidal activity was associated with increased metabolic activity in the remaining cells, as verified by ATP analysis.

**Figure 5:**
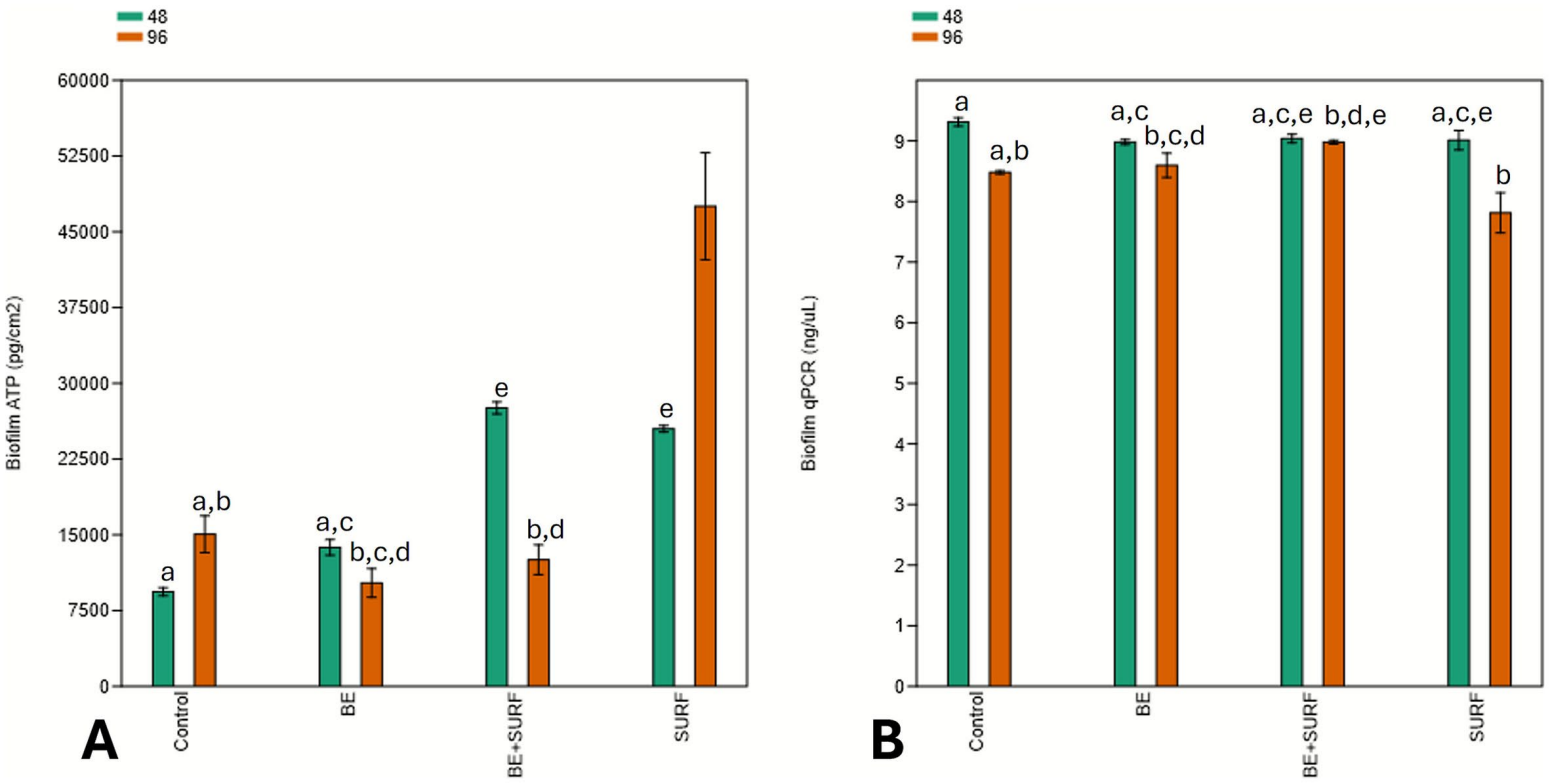
Biofilm ATP (pg/cm^2^) **(A)** and cellular concentration (ng/μL) **(B)** over time (48 and 96 h) under different treatment conditions: bioemulsifier (BE), surfactin (SURF), bioemulsifier + surfactin (BE+SURF), and control. Treatments labeled with the same letter do not differ statistically (*p* ≤ 0.05).

ATP analysis of the biofilms revealed increased metabolic activity in microorganisms treated with the bioemulsifier combined with surfactin at 48 h and with surfactin alone at both 48 and 96 h compared with the control (Figure 5A). The bioemulsifier alone did not significantly differ from the control. Although some treatments resulted in statistically significant differences in cellular metabolism, the total cell concentration did not vary significantly across most treatments over time, as shown in Figure 5B (qPCR). Significant differences were observed only between the bioemulsifier (96 h), the bioemulsifier combined with surfactin (96 h), and surfactin (48 h) compared with surfactin at 96 h, which presented the lowest cell concentration. In this case, surfactin was the treatment responsible for reducing the number of adhered cells, indicating that biofilm disruption was associated with increased metabolic activity in the remaining cells, as evidenced by ATP analysis.

**Figure 6:**
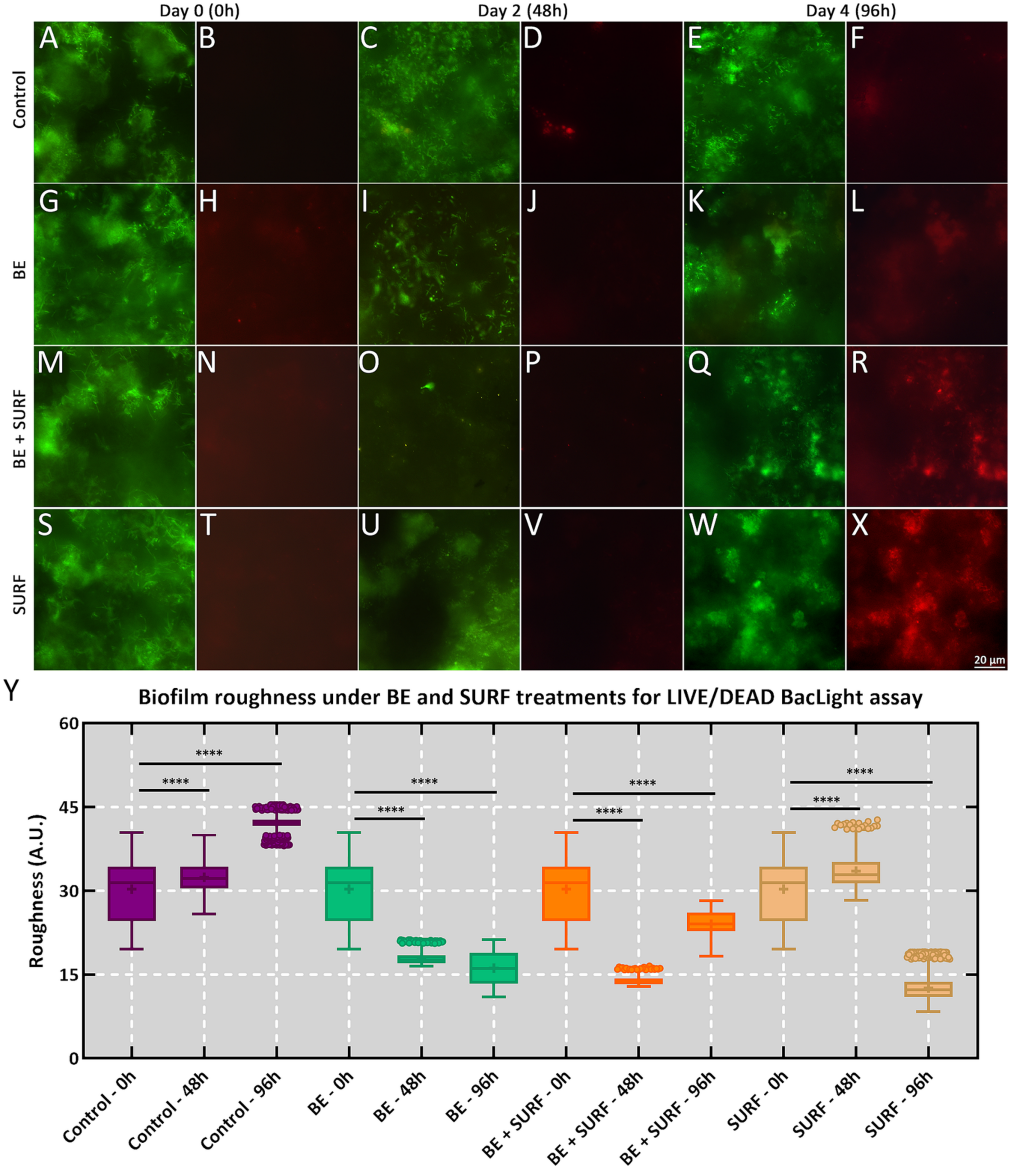
Analysis of microbial biofilms on metallic coupons via a live/dead BacLight assay via epifluorescence microscopy. The samples were monitored for up to 96 h (day 4). **(A–F)** Control conditions. **(G–L)** Samples treated with BE. **(M–R)** Samples treated with BE+SURF. **(S–X)** Samples treated with SURF. **(Y)** Boxplot of biofilm roughness from samples treated with BE, BE+SURF, and SURF against the control condition. For each condition, the left panel reflects Syto9-stained cells (i.e., live), whereas the right panel reflects PI-stained cells (i.e., dead). Note that **** represents statistical inference at a significance level of *p* < 0.0001.

Regarding biofilm formation on the test samples, the topographic roughness profile—obtained via epifluorescence microscopy to illustrate the cellular viability of biofilms adhered to metal coupons within the bioreactors—revealed that the control group exhibited a progressive increase in surface roughness over time, with mean values of 30.30 ± 5.76 A. U. (0 h), 32.40 ± 3.30 A. U. (48 h), and 42.18 ± 1.40 A. U. (96 h), indicating continuous growth of the microbial extracellular matrix. The differences between time points were highly significant (****p < 0.0001), reflecting structural maturation of the biofilm (Figure 6). Treatments with the bioemulsifier and the bioemulsifier + surfactin combination resulted in the lowest roughness values, suggesting a significant impact on biofilm structural organization. The bioemulsifier treatment yielded mean roughness values of 17.99 ± 1.17 A. U. (48 h) and 16.13 ± 2.87 A. U. (96 h), both of which were significantly lower than those of the control (****p < 0.0001). These findings suggest that the bioemulsifier reduces extracellular matrix cohesion, impairing the maintenance of the biofilm’s three-dimensional structure. The combined bioemulsifier and surfactin treatment had similar effects, with roughness values of 13.86 ± 0.77 A. U. (48 h) and 24.05 ± 2.46 A. U. (96 h), respectively. Although the roughness slightly increased at the end of the experiment, it remained statistically lower than that of the control (****p < 0.0001). The synergistic action of these compounds may be related to surfactin’s ability to destabilize the interface between cells and the extracellular matrix, increasing the susceptibility of biofilms to mechanical removal. The treatment with surfactin alone had the most pronounced effect on reducing roughness, reaching 12.62 ± 2.43 A. U. at 96 h, which was significantly lower than that of the control (****p < 0.0001).

**Figure 7:**
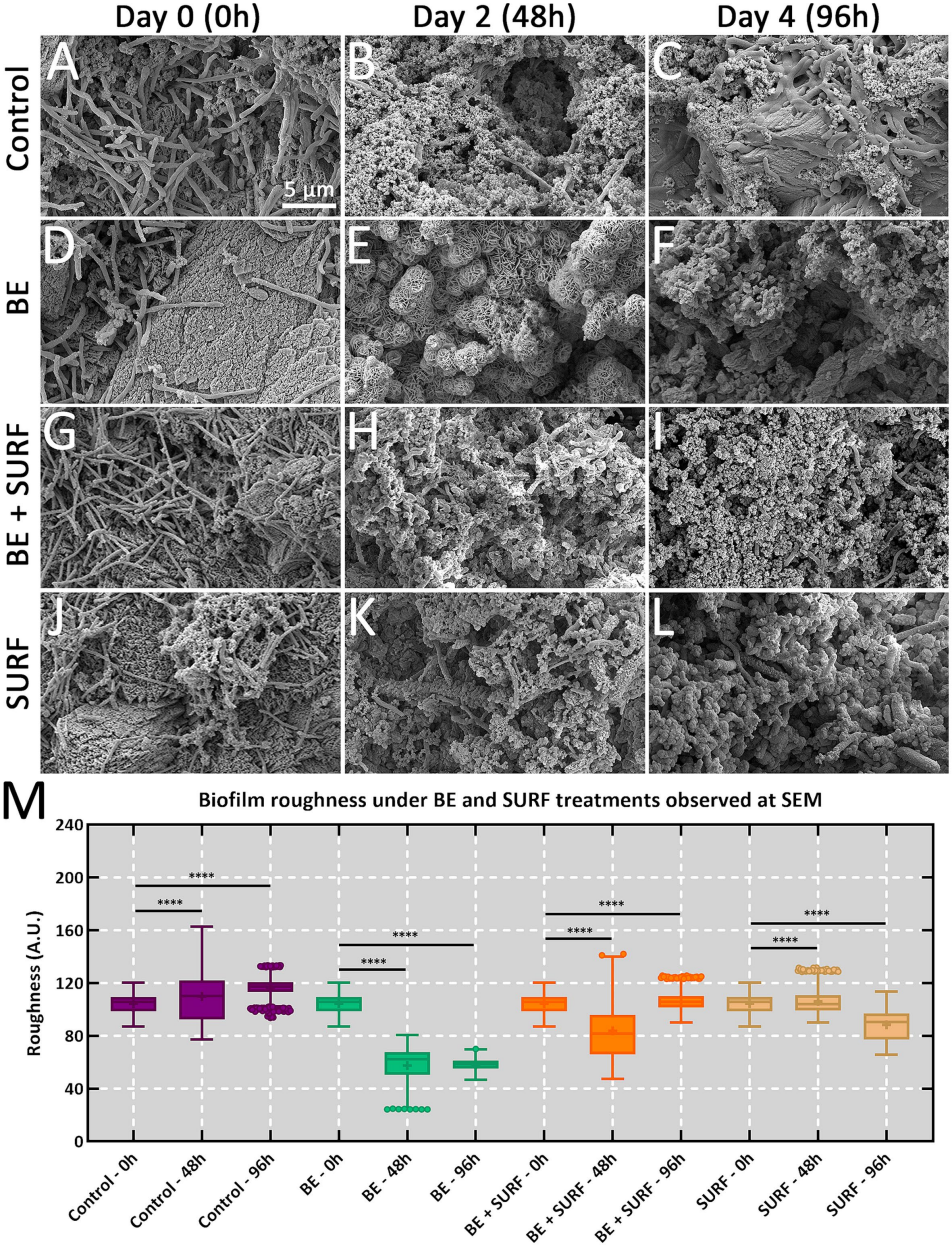
SEM analysis of microbial biofilms on metallic coupons. The samples were monitored for up to 96 h (4 days). **(A–C)** Control conditions. **(D–F)** Samples treated with BE. **(G–I)** Samples treated with BE+SURF. **(J–L)** Samples treated with SURF. **(M)** Boxplot of biofilm roughness from samples treated with BE, BE+SURF, and SURF against the control condition. Note that **** represents statistical inference at a significance level of *p* < 0.0001.

The results obtained through scanning electron microscopy revealed significant alterations in the roughness of the microbial biofilm in response to the different treatments. The control group exhibited a progressive increase in roughness over time, with mean values of 104.5 ± 7.13 nm at 0 h, 109.9 ± 19.00 nm at 48 h, and 116.4 ± 7.17 nm at 96 h, corroborating the findings from epifluorescence microscopy regarding cellular viability. Statistical analysis revealed significant differences among all control measurements (****p < 0.0001), indicating natural biofilm growth over time (Figure 7).

The bioemulsifier also significantly reduced biofilm roughness. The roughness decreased from 104.5 ± 7.13 nm at 0 h to 58.01 ± 4.62 nm after 96 h (****p < 0.0001). The combination of the bioemulsifier and surfactin resulted in an initial significant reduction, with the roughness reaching 83.70 ± 21.95 nm at 48 h; however, a slight increase was observed at 96 h (105.9 ± 6.22 nm), suggesting a possible adaptation of the biofilm to the compound combination. Treatment with surfactin alone also resulted in a reduction in biofilm roughness, from 104.5 ± 7.13 nm at 0 h to 88.37 ± 11.93 nm at 96 h, with statistically significant differences (****p < 0.0001). Nevertheless, this reduction was less pronounced than that resulting from the exclusive use of the bioemulsifier.

The slight increase in biofilm roughness at 96 h with the combined bioemulsifier and surfactin treatment reflects stress-response adaptation rather than full structural recovery (Figures 5A, 6, 7). The ATP peak at 48 h suggested transient metabolic activation for matrix repair (EPS/eDNA deposition), even without a proportional increase in sessile cell counts (Figures 5B, 7). SEM analysis revealed only a modest increase in roughness, which remained well below control levels, indicating partial matrix densification but not a return to pretreatment architecture. The mass loss did not decrease at 96 h with the mixture, and surfactin alone was the most effective at 96 h (Figure 2).

**Figure 8:**
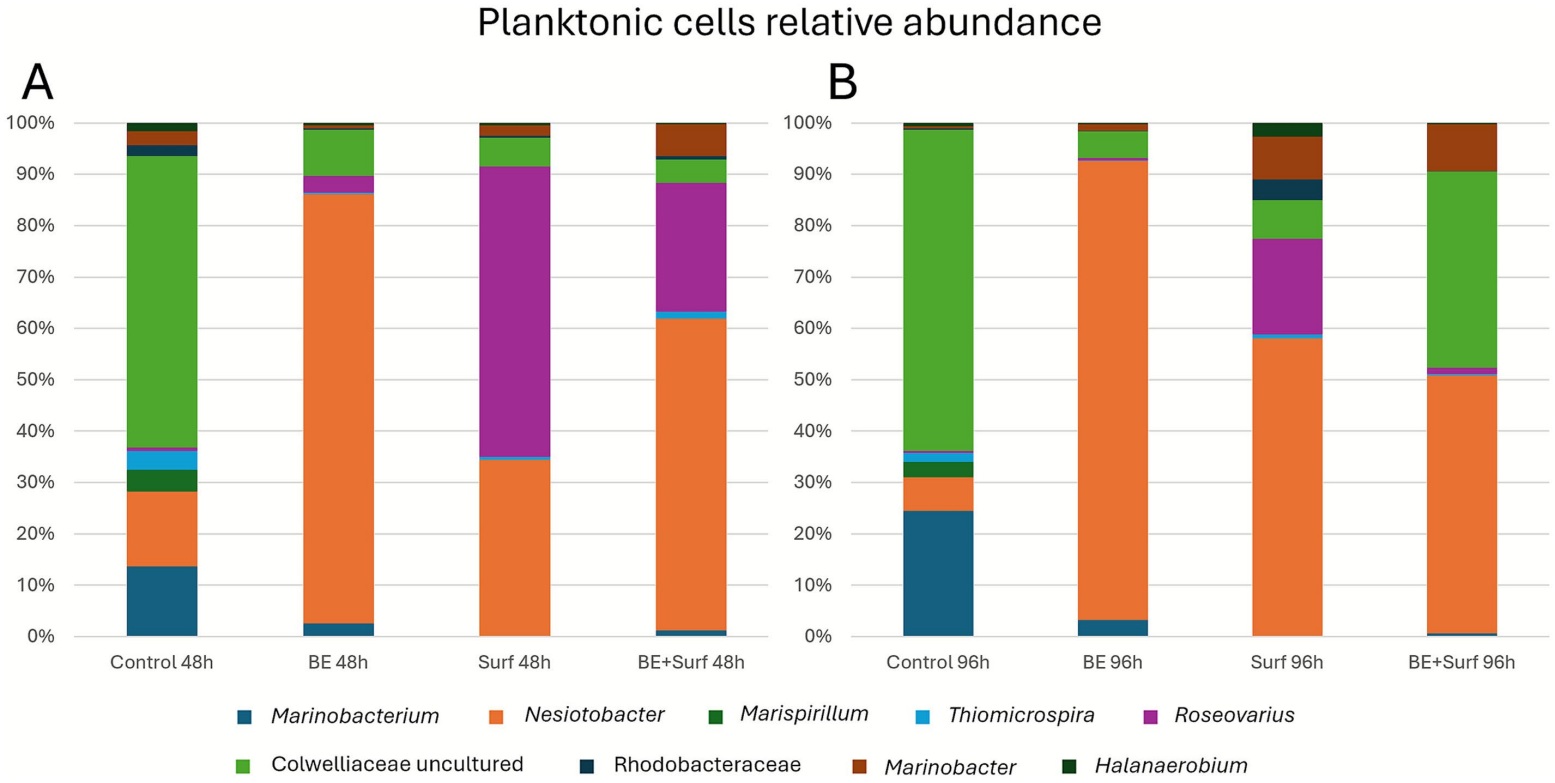
Relative abundance of the genera found in the planktonic form at 48 h **(A)** and 96 h **(B)** for the control and each treatment: bioemulsifier (BE), surfactin (SURF), and bioemulsifier + surfactin (BE+SURF).

**Figure 9:**
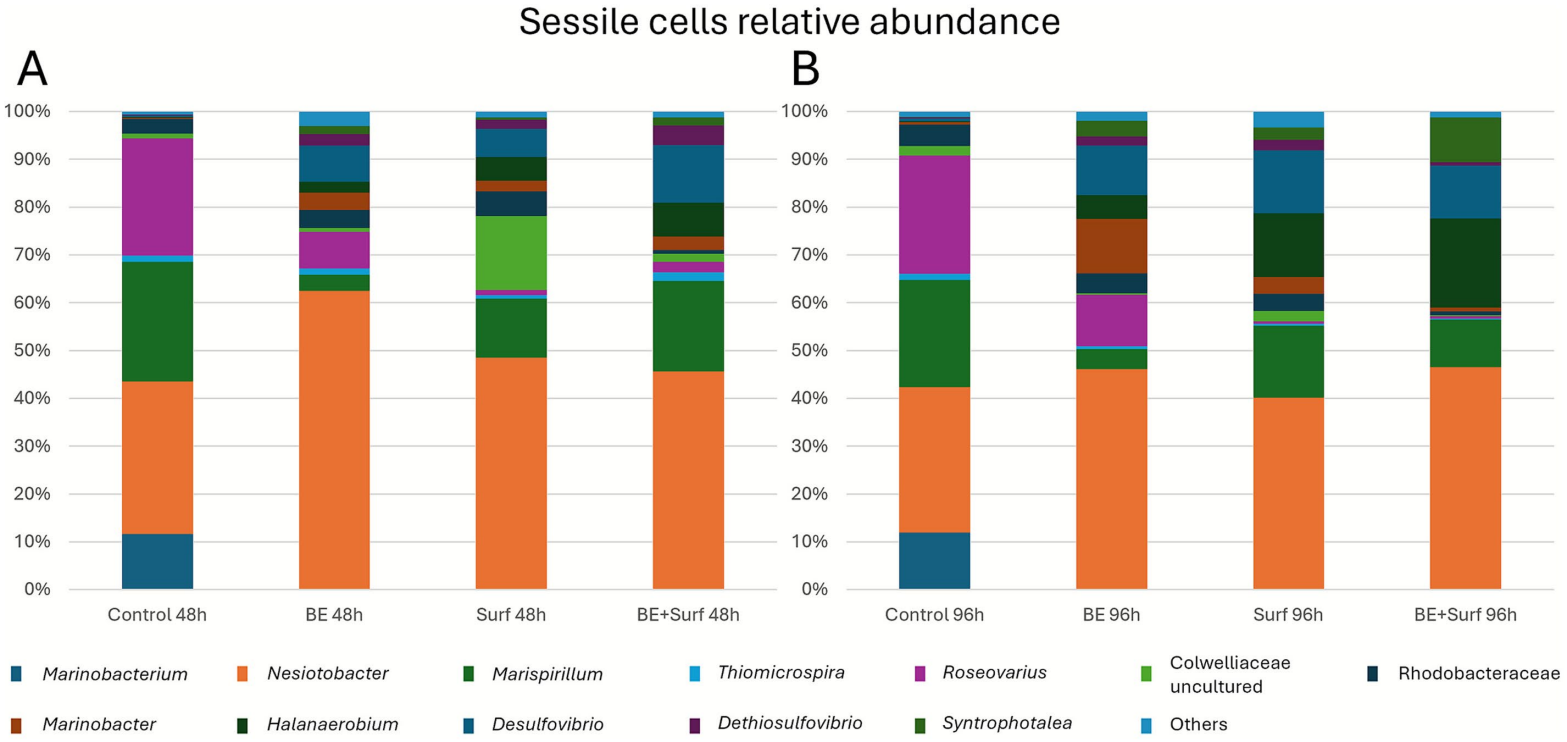
Relative abundance of the genera found in the sessile form (adhered cells/biofilms) at 48 h **(A)** and 96 h **(B)** for the control and each treatment: bioemulsifier (BE), surfactin (SURF), and bioemulsifier + surfactin (BE+SURF).

Mechanistically, two processes are likely: (i) community rebalancing, with enrichment of less aggressive taxa (e.g., *Nesiotobacter*) and depletion of corrosive groups (SRB/MOB) in absolute terms, although *Marinobacter* and *Desulfovibrio* persist in some fractions (Figures 8, 9 and Supplementary Figures S3, S4); (ii) matrix remodeling, as surfactin weakens cell–matrix adhesion and the bioemulsifier disrupts cohesion, leading to stress-induced EPS synthesis and a slight increase in roughness, which is still much lower than that of the controls. qPCR revealed no significant biomass increase at 96 h (Figures 4B, 5B), and corrosion protection remained (Figure 2), indicating that the rebound was due to adaptive matrix packing, not regrowth of a corrosive consortium.

According to the results of the Kruskal–Wallis pairwise analysis, the phylogenetic diversity of the planktonic microorganisms was significantly distinct from that of the biofilm communities (*p* < 0.000025). All the treatments resulted in significant differences from the control in both the biofilm (*p* < 0.01) and planktonic (*p* < 0.001) forms.

#### 3.2.1 Microbial community quantification and characterization

Beta diversity analyses supported by the PERMANOVA test revealed significant differences in species composition across all treatment combinations (*p* < 0.05). The PCoA plot illustrating these differences is provided in the Supplementary Figure S2. 16S rRNA sequencing and absolute quantification revealed a shift in the microbial community structure, with a reduced abundance of corrosion-associated taxa such as sulfate-reducing and metal-oxidizing bacteria (Supplementary Figures S3, S4).

16S rRNA sequencing revealed the relative abundances of genera in the planktonic and sessile (adhered cells/biofilm) communities at 48 h and 96 h for the control and each treatment, as shown in Figures 8, 9, respectively. In general, among the planktonic taxa identified at lower taxonomic levels (Figure 8), the most abundant were uncultured genera from the Colwelliaceae family, *Nesiotobacter*, *Marinobacterium*, *Marispirillum*, *Thiomicrospira*, *Marinobacter*, members of the Rhodobacteraceae family, *Halanaerobium*, and *Roseovarius*, among others.

The dominant planktonic genus in the control samples at both 48 and 96 h was the uncultured genus from the Colwelliaceae family (49.1 and 52.2%, respectively). In contrast, in the treatments with the bioemulsifier (73.9 and 74.9% at 48 and 96 h, respectively), surfactin at 96 h (52.2%), and the bioemulsifier combined with surfactin (50.1 and 43.4% at 48 and 96 h, respectively), the dominant genus shifted to *Nesiotobacter*. The only exception was the surfactin treatment at 48 h, in which *Roseovarius* was predominant (55%), followed by *Nesiotobacter* (33.3%). After 96 h of treatment, both surfactin and the bioemulsifier combined with surfactin resulted in an increase in *Marinobacter* (7.4 and 7.9%, respectively) compared with the control and the 48-h time point. *Halanaerobium* (2.4%) and Rhodobacteraceae (3.7%) also increased in response to surfactin treatment relative to the other conditions. Compared with that of the control, the abundance of *Marinobacterium* substantially decreased across all the treatments (ranging from 0 to 2.7%), which initially reached 11.8 and 20.4% at 48 and 96 h, respectively. Similarly, *Marispirillum* was virtually absent in all the treatments, whereas it was present in 3.7 and 1.4% of the control samples at 48 and 96 h, respectively.

Among the sessile genera identified (Figure 9), the most abundant taxa included *Nesiotobacter*, *Marispirillum*, *Roseo*var*ius*, *Marinobacterium*, members of the Rhodobacteraceae family, an uncultured genus from the Colwelliaceae family, and *Thiomicrospira*, *Desulfovibrio*, *Marinobacter*, *Halanaerobium*, *Dethiosulfovibrio*, and *Syntrophotalea*, among others. The results revealed that *Nesiotobacter* was the dominant genus across all the samples at both 48 and 96 h, accounting for between 30.4 and 62.5% of the total abundance. All three bioproduct treatments led to an increase in *Nesiotobacter* relative to the control (32.0 and 30.4% at 48 and 96 h, respectively). The bioemulsifier alone resulted in the highest abundance of *Nesiotobacter* (62.0 and 46.1% at 48 and 96 h), whereas surfactin alone and the combined bioemulsifier–surfactin treatment yielded similar levels, approximately 45.2%.

In contrast, the treatments markedly reduced the abundance of *Marinobacterium*, which declined from 12.0% in the control to values ranging between 0.0 and 0.1%. A similar trend was observed for *Marispirillum*, which decreased from approximately 23.7% in the control to between 3.4 and 18.9%, depending on the treatment. *Roseovarius* also showed a substantial reduction, with its relative abundance decreasing from 24.6% in the control to between 0.4 and 10.8% under the treatment conditions. Conversely, the treatments promoted the growth of other genera, including *Desulfovibrio*, *Halanaerobium*, *Marinobacter*, and *Syntrophotalea*.

## 4 Discussion

### 4.1 Production and physicochemical properties

The physicochemical characteristics of the surfactin produced by *Bacillus velezensis* H2O-1 were consistent with previously reported findings for this strain (Guimarães et al., 2019, 2021; de Paula Vieira de Castro et al., 2022). These results reinforce the reproducibility of surfactin production under controlled cultivation conditions and validate its application as an effective biosurfactant. Key parameters such as surface tension reduction, emulsification index, and critical micelle concentration aligned closely with those described in the literature, confirming the functional integrity of the compound.

To be classified as an effective biosurfactant, a compound must demonstrate the ability to reduce the water surface tension from approximately 72.8 mN/m to values below or near 35 mN/m (Sharma, 2016). The bioproduct produced by *Psychrobacillus antarcticus* Val9 achieved a surface tension of 45.23 ± 1.6 mN/m and exhibited a strong capacity to form stable emulsions across a range of conditions, including various oils, pH values, and temperatures. Given its limited surface tension reduction yet pronounced emulsifying activity, the compound is more accurately classified as a bioemulsifier.

### 4.2 Antimicrobial, antibiofilm, and anticorrosion effects

Our results show that simply dosing injection-header water with our *Psychrobacillus*-derived bioemulsifier and surfactin markedly curbed carbon-steel weight loss—an effect that was both rapid and sustained over 96 h. In light of the staggering global corrosion burden—estimated at approximately US$ 2.5 trillion annually, with MIC driving at least 20% of that—the observed mass-loss reduction is more than a laboratory curiosity (Tuck et al., 2022). Thus, it could represent a scalable, greener approach to corrosion control that complements or even surpasses conventional biocides. Whereas weekly glutaraldehyde pulses in mixed-species reactors merely blunted sulfide peaks before communities rebound (Jones et al., 2025), our bioproducts delivered continuous surface protection without the typical “rebound” corrosion rates observed when biofilms persist (Liu et al., 2023; Lyu et al., 2024).

The low mass loss observed after 96 h in the presence of surfactin is indeed expected and consistent with the physicochemical and biological properties of this biosurfactant. Surfactin is well-known for its ability to reduce surface and interfacial tension, disrupt biofilm formation, and inhibit the activity of corrosion-associated microorganisms. Specifically, surfactin destabilizes the extracellular matrix of biofilms, making it more difficult for corrosion-promoting microorganisms to adhere and form mature biofilms. Following this disruption, surfactin may also form a protective layer on the metal surface, reducing direct contact between corrosive agents and the alloy (Guimarães et al., 2019; de Paula Vieira de Castro et al., 2022).

Additionally, our results show that the biosurfactant selectively suppressed key taxa involved in MIC, such as sulfate-reducing and metal-oxidizing bacteria, as confirmed by both qPCR and 16S rRNA sequencing. The treatments also influenced the overall microbial community composition, as shown in Supplementary Figures S3, S4, where several bacterial genera presented reduced absolute and relative abundances. These findings support the conclusion that corrosion mitigation involves both physicochemical protection and biological inhibition mechanisms. Together, these effects explain the stabilization of corrosion and the remarkably low mass loss after 96 h, indicating the establishment of a protective equilibrium rather than experimental variability.

At the cellular level, ATP assays and qPCR revealed an interplay between biocidal action and community metabolism. Bulk ATP in our bioemulsifier treatments increased modestly, suggesting stress-induced hyperactivity among survivors, whereas total cell counts decreased, which was consistent with selective killing rather than broad-spectrum inhibition. This phenomenon is consistent with literature reports in the literature, where antimicrobial or surfactant exposure can trigger a compensatory increase in energy metabolism as cells attempt to maintain homeostasis and repair damage (Li and Zhu, 2012; Zhang et al., 2023). This pattern contrasts sharply with dual-reactor studies under glutaraldehyde, where ATP and sulfide signals are repeatedly spiked, resulting in only partial microbial suppression (Tripathi et al., 2021; Jones et al., 2025). Importantly, this ATP surge occurred alongside a reduction in total cell counts (as measured by qPCR), suggesting selective killing and metabolic activation of the remaining population, rather than broad-spectrum inhibition or uncontrolled proliferation. The observed ATP increase, coupled with reduced biofilm roughness and cell density, supports the hypothesis of stress-induced metabolic compensation rather than enhanced growth. By chipping away at cell numbers without provoking complete metabolic rebound, our formulations appear to destabilize biofilm homeostasis—a common weak spot that conventional chemicals often miss (Yang et al., 2024). Additionally, observations via SEM and epifluorescence microscopy reinforced these findings. Both techniques revealed that the treated coupons bore far smoother, more uniform surfaces and thinner, patchier biofilms. The surface-roughness metrics plummet to near-baseline values in the bioemulsifier and surfactin groups, whereas the controls thickened steadily into rough, rusticle-like architectures characteristic of sulfate-reducing consortia on steel (Xu et al., 2023; Didouh et al., 2024). The diminished extracellular matrix cohesion we observed likely reflects disrupted EPS scaffolding, making biofilm cells more prone to detachment under shear—a stark departure from the entrenched, pit-prone films described in marine and oilfield contexts. Finally, our community profiling highlights the broader ecological impact: treatments shifted taxonomic profiles toward less corrosive and lower-biomass assemblages. In contrast, mixed-species reactors treated with glutaraldehyde often select for stress-tolerant extremophiles that maintain robust attachment (dos Santos et al., 2022; Jones et al., 2025), perpetuating corrosion cycles. Thus, by reducing overall cell density and hampering EPS stabilization, our bioproducts foster a microbial environment that is unfavorable for sustained metal dissolution. In summary, these findings underscore how targeted biological agents can not only outpace traditional chemical methods for reducing mass loss but also strategically rewire biofilm structure and community dynamics to yield longer-lasting anticorrosion solutions.

#### 4.2.1 Microbial community composition and structural dynamics through relative and absolute abundances

Our sequencing results revealed that members of the Colwelliaceae family were predominant in control planktonic samples at both 48 and 96 h. Members of this family are widely distributed in marine environments and are known to participate in the decomposition of diverse organic substrates, including hydrocarbons, lipids, proteins, and polysaccharides (Lee et al., 2025). Knapik et al. (2019) reported a marked increase in the abundance of Colwelliaceae in seawater samples, both with and without oil exposure, following the lysis of algae and diatom cells and an increase in organic matter in the environment. These dynamics suggest that this family harbors genera with broad metabolic capabilities, which may explain their predominance in controls. However, under the altered conditions imposed by the treatments, other taxa appear to have been favored, leading to a reduction in Colwelliaceae dominance (Knapik et al., 2019). This trend was consistent with the absolute quantification via qPCR (Supplementary Figure S3), which also revealed a marked decrease in the copy number of Colwelliaceae under the different treatments.

The application of the bioemulsifier, surfactin, and their combination resulted in an enrichment in the relative abundance of the genus *Nesiotobacter*, which became the most dominant group in all planktonic treatment samples, except for surfactin at 48 h. *Nesiotobacter* was first described by Donachie et al. (2006) from a hypersaline lake (Donachie et al., 2006) and remains poorly explored, with only two species currently described (Park et al., 2025) still, emerging evidence points to its metabolic versatility and biotechnological relevance, particularly in hydrocarbon degradation and biosurfactant production. For example, *Nesiotobacter* has been detected in deep-sea sediments and has been shown to degrade toluene under high-pressure conditions, with genomic traits supporting biosurfactant production (Ganesh Kumar et al., 2019). In another study, an increase in *Nesiotobacter* abundance was observed in the presence of zinc oxide, a compound used to mitigate corrosion by inhibiting SRB (Jafari et al., 2024).

In sessile communities, *Nesiotobacter* was also dominant at both 48 and 96 h, with higher relative abundances in all the treatments than in the control, and the greatest enrichment occurred under bioemulsifier addition. The dominance of *Nesiotobacter* in sessile communities, despite not being typically described as biofilm-forming, is consistent with the ecological versatility highlighted in the previous paragraph, which may provide a competitive advantage under altered physicochemical conditions. However, it should be noted that this enrichment was observed at the relative level; absolute quantification indicated that *Nesiotobacter* copy numbers generally decreased under the various treatments, suggesting that its apparent dominance reflects community rebalancing, with *Nesiotobacter* persisting while other taxa declined more sharply.

In addition, some other abundant genera identified for planktonic and sessile communities are recognized in the literature as being related to MIC processes, such as metal-oxidizing bacteria (e.g., *Marinobacter* and *Mariprofundus*) and sulfur-oxidizing bacteria (e.g., *Thiomicrospira*). Among the sulfate-reducing bacteria, *Desulfovibrio* and *Dethiosulfovibrio* were both detected in sessile communities (Liu et al., 2023).

Although amplicon sequencing suggested enrichment of *Desulfovibrio* and *Dethiosulfovibrio* in the treated samples, absolute quantification by qPCR revealed that these taxa presented decreased copy numbers compared with those of the control. SRB, particularly *Desulfovibrio*, are recognized as key agents driving long-term MIC (Liu et al., 2024); however, microbial corrosion is not driven by a single species in isolation but by dynamic successions in biofilm communities, where metabolic activities such as hydrogen sulfide production, pH reduction, and electron transfer are tightly interlinked (Liu et al., 2023). The difference we found between relative and absolute abundance highlights the complexity of microbial interactions and illustrates that shifts in relative abundance do not necessarily reflect true population growth but rather changes in the balance of the whole community. Moreover, the reduction in the number of SRB under the surfactin, bioemulsifier, and combined treatments highlights the efficacy of the tested bioproducts in suppressing the microorganisms most directly associated with the MIC.

These findings suggest that the tested bioproducts not only reduce SRB abundance but also disrupt biofilm structure and community homeostasis, making it more difficult for residual *Desulfovibrio* or other SRB to regain dominance. While a significant reduction in both the relative and absolute abundances of *Desulfovibrio* and other SRB taxa was observed after the application of the bioemulsifier and surfactin, trace populations were still detectable. To mitigate the risk of the rebound effect, a scheduled, continuous, or pulsed application of the bioproducts could be implemented. Regular dosing would help maintain microbial suppression, prevent biofilm maturation, and avoid conditions that allow SRB populations to recover and re-establish corrosive consortia.

## 5 Conclusion

The striking reduction in carbon-steel mass loss—down by more than 50% in some treatments after 96 h—highlights the real-world potential of our *Psychrobacillus*-derived bioemulsifier and *Bacillus velezensis* surfactin. By achieving a 63 ± 1.7% emulsification index at 96 h and lowering the surface tension to 28.9 mN/m (respectively), these bioproducts disrupted the usual formation of thick, rough biofilms (e.g., in controls, which climbed to a roughness of 42.2 ± 1.4 A. U.), restoring surfaces to near-baseline smoothness (e.g., as low as 12.6 ± 2.4 A. U. with surfactin alone). Unlike conventional glutaraldehyde regimens—which often trigger transient metabolic rebounds and the rise of stress-tolerant taxa—our formulations drove down total cell counts by up to 40%, blunted ATP surges in the bulk and biofilm phases, and prevented the characteristic ‘rusticle’ architectures of sulfate-reducer consortia.

Beyond sheer efficacy, this bioproduct suite rewires community dynamics, as qPCR and 16S rRNA gene sequencing revealed a shift from corrosion-prone assemblages to less aggressive, low-biomass populations, cutting the habitat–engineering feedback loops that fuel MIC. Coupled with the remarkable thermal and pH stability of the bioemulsifier—maintaining ~60% emulsification under extremes and increasing performance to 76% after autoclaving—these findings point to a robust, environmentally friendly toolkit for industries ranging from offshore pipelines to water-injection systems. By integrating targeted microbial disruption with surface protection, our approach offers a sustainable path forward where durability, ecological safety, and long-term corrosion control must coexist.

## Data Availability

The raw sequencing data generated for this study have been deposited in the NCBI Sequence Read Archive (SRA) under BioProject accession number PRJNA1322041. The dataset is publicly available at: https://www.ncbi.nlm.nih.gov/bioproject/PRJNA1322041.

## Footnotes

1
http://novogene.egnyte.com

2
https://earthmicrobiome.org/protocols-and-standards/16s/
