# Supplementary material for: Anticorrosion potential of a bioemulsifier produced by Psychrobacillus antarcticus Val9 isolated from Antarctic soil
Source: Front Microbiol. 2026 Jan 5;16:1694832. doi: 10.3389/fmicb.2025.1694832 (PMC12812664; doi:10.3389/fmicb.2025.1694832)
Supplement: Supplementary file 1 [file Data_Sheet_1.docx]

**Anticorrosion potential of the bioemulsifier produced by *Psychrobacillus antarcticus* Val9 isolated from Antarctic soil**

Lívia Vieira Araujo de Castilho, Aline Loureiro Barreto, Karen Caroline Ferreira Santaren, Mariana Barbalho Farias Rosenblatt, Igor Taveira, Jefferson Cypriano, Fernanda Abreu, Mateus Gomes de Godoy, Diogo de Azevedo Jurelevicius, Lucy Seldin

**SUPPLEMENTARY MATERIAL**


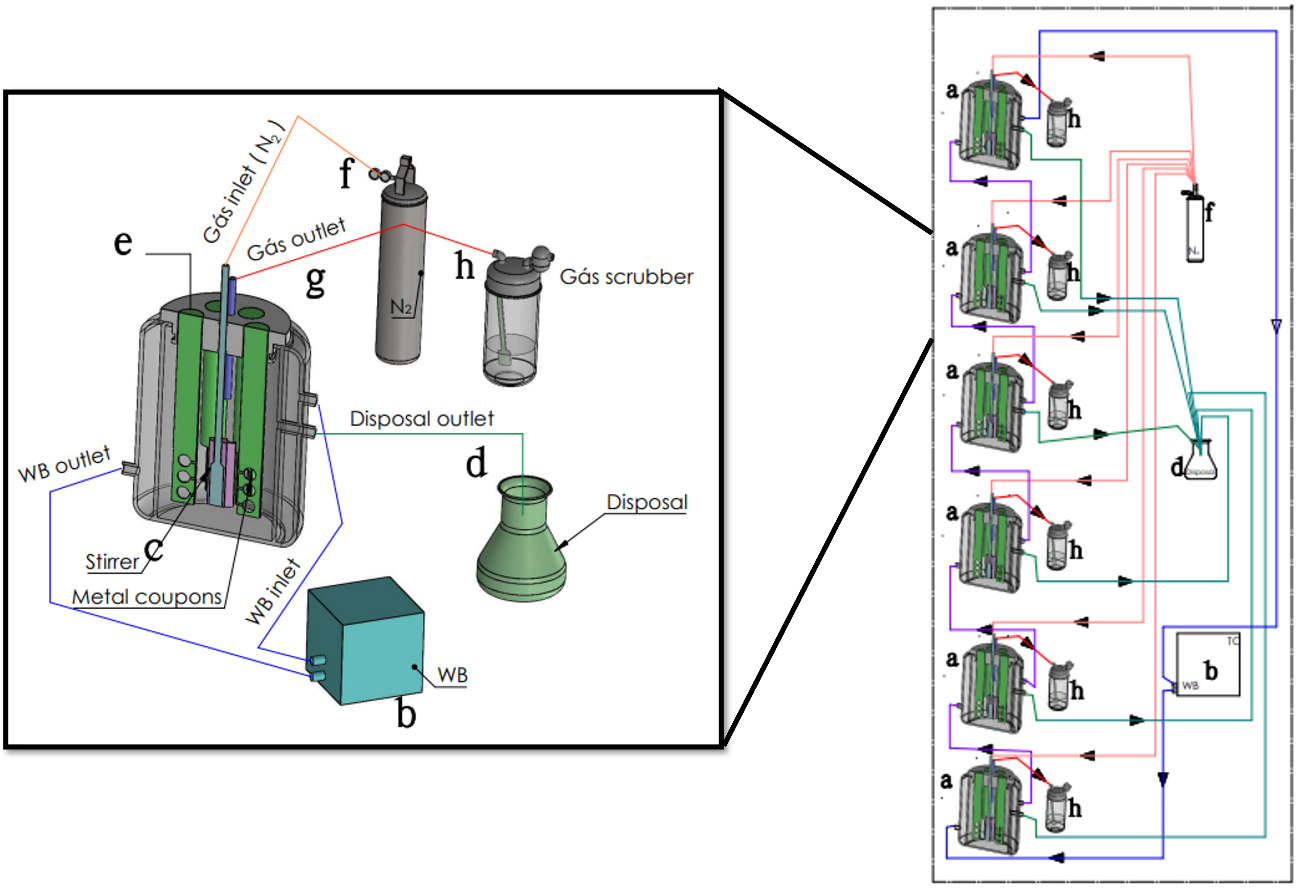


**Figure S1.** Custom-designed bioreactors comprising: (a) jacketed borosilicate glass

bioreactors coupled in series; (b) temperature control via a recirculation system

connected to an external water bath-WB; (c) internal agitation coupled with a magnetic stirring platform; (d) lateral outlet for bulk disposal connected by tubing to an Erlenmeyer flask; (e) openings fitted with rods to support triplicate samples of metal coupons; (f) Nitrogen injection; (g) sample collection/biocides injection; (h) gas venting coupled to a gas scrubber.


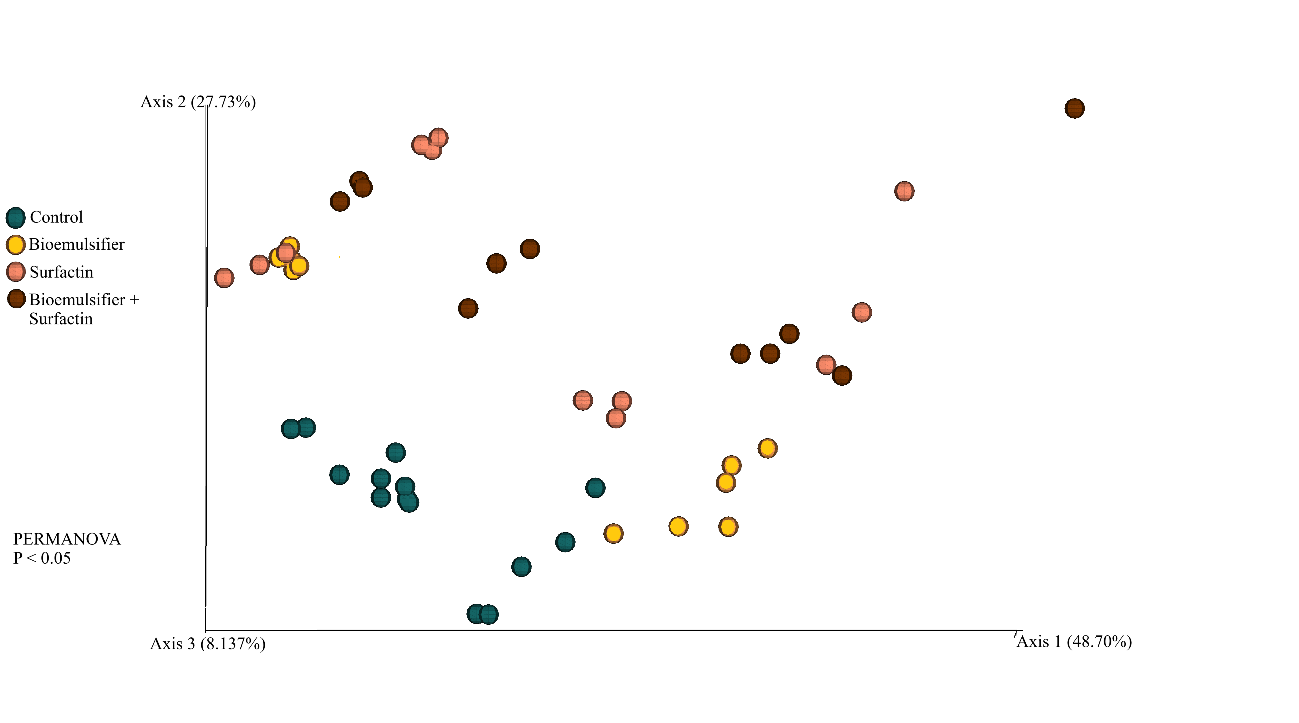


**Figure S2.** Principal Coordinates Analysis (PCoA) based on weighted UniFrac distances of bacterial communities. Each point represents one sample, colored according to treatment (Control (green); bioemulsifier (yellow), surfactin (pink), bioemulsifier + surfactin (brown)).


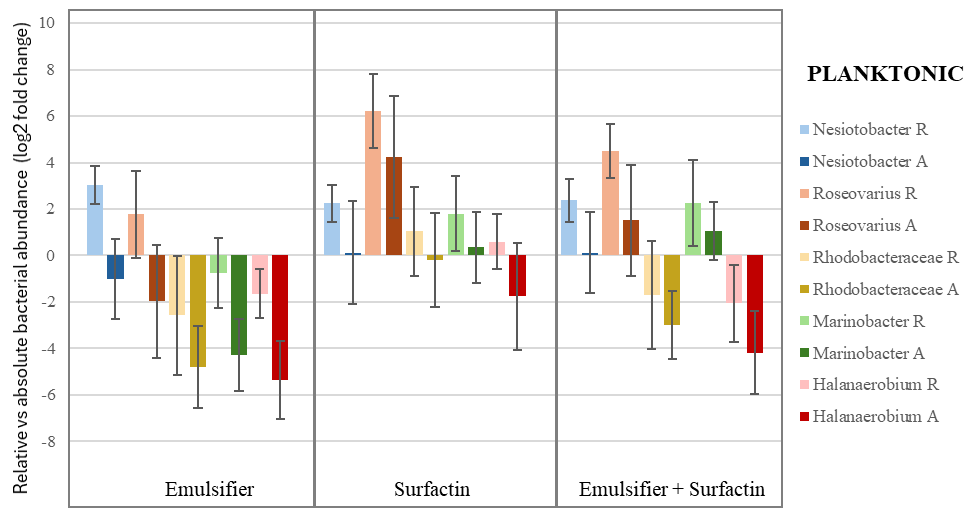


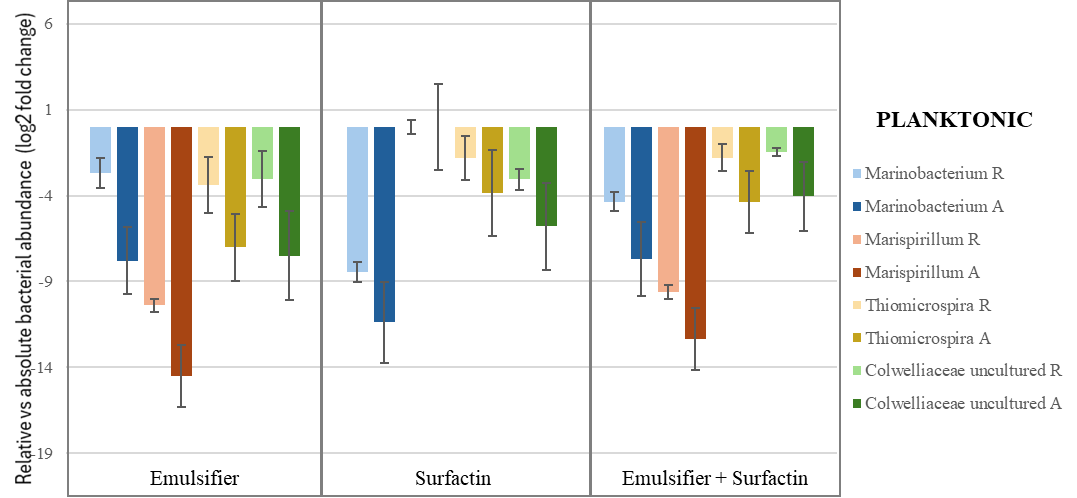


**Figure S3.** Relative and absolute log₂ fold changes of selected bacterial taxa in planktonic communities after treatment with bioemulsifier (Emulsifier), surfactin, or their combination (Emulsifier + Surfactin). Bars represent mean log₂FC compared to the control, with error bars indicating standard deviation (n=2). ‘R’ denotes relative abundance, while ‘A’ denotes absolute abundance.


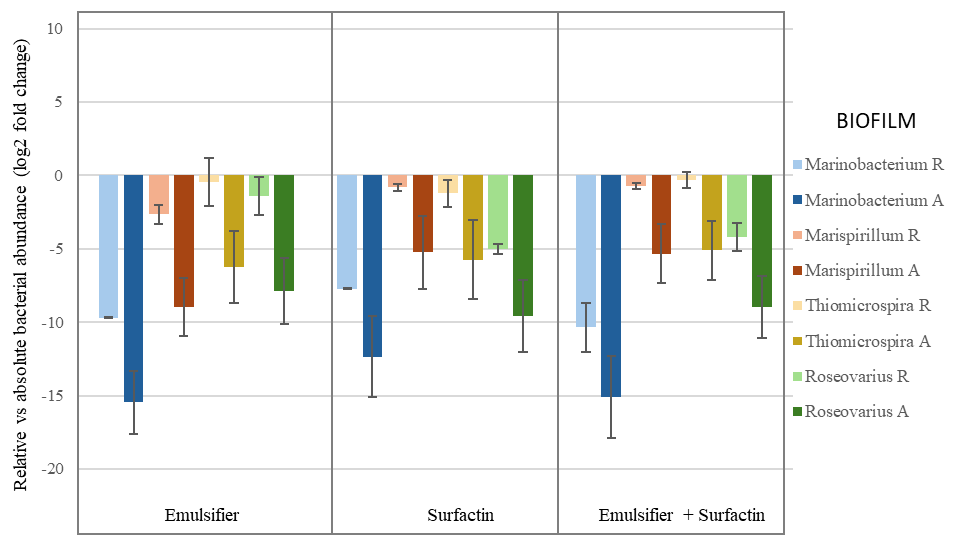


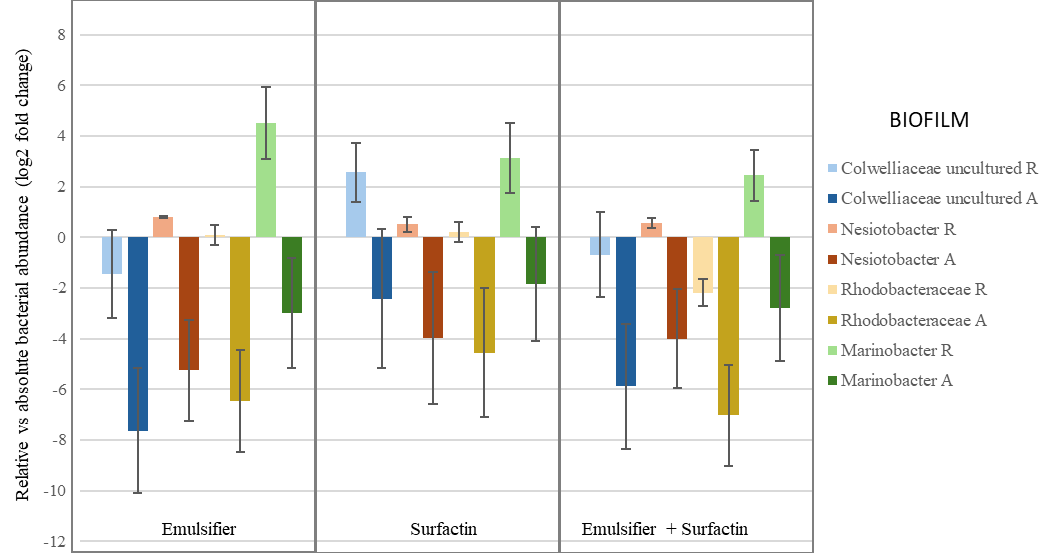


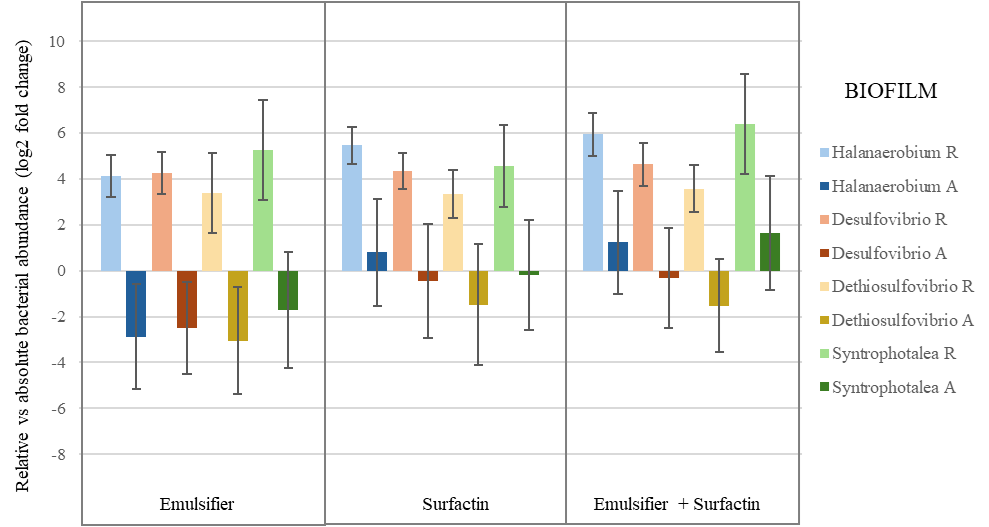


**Figure S4.** Relative and absolute log₂ fold changes of selected bacterial taxa in biofilm communities after treatment with bioemulsifier (Emulsifier), surfactin, or their combination (Emulsifier + Surfactin). Bars represent mean log₂FC compared to the control, with error bars indicating standard deviation (n=2). ‘R’ denotes relative abundance, while ‘A’ denotes absolute abundance.
